# Vital Members in the More Dysbiotic Oropharyngeal Microbiotas in H7N9-Infected Patients

**DOI:** 10.3389/fmed.2020.00396

**Published:** 2020-08-11

**Authors:** Hua Zha, Haifeng Lu, Jieyun Wu, Kevin Chang, Qiangqiang Wang, Hua Zhang, Jinyou Li, Qixia Luo, Yanmeng Lu, Lanjuan Li

**Affiliations:** ^1^State Key Laboratory for Diagnosis and Treatment of Infectious Disease, Collaborative Innovation Center for Diagnosis and Treatment of Infectious Diseases, National Clinical Research Center for Infectious Diseases, The First Affiliated Hospital, College of Medicine, Zhejiang University, Hangzhou, China; ^2^School of Biological Sciences, The University of Auckland, Auckland, New Zealand; ^3^Institute of Marine Science, The University of Auckland, Auckland, New Zealand; ^4^Plant Health and Environment Laboratory, Ministry for Primary Industries, Auckland, New Zealand; ^5^Department of Statistics, The University of Auckland, Auckland, New Zealand; ^6^Department of Geriatrics, The First Affiliated Hospital, College of Medicine, Zhejiang University, Hangzhou, China

**Keywords:** H7N9, microbial colonization states, oropharyngeal microbiota, dysbiosis status, secondary bacterial lung infection, illumina sequencing

## Abstract

The dysbiosis of oropharyngeal (OP) microbiota is associated with multiple diseases, including H7N9 infection. Different OP microbial colonization states may reflect different severities or stages of disease and affect the effectiveness of the treatments. Current study aims to determine the vital bacteria that could possibly drive the OP microbiota in the H7N9 patients to more severe microbial dysbiosis state. The OP microbiotas of 42 H7N9 patients and 30 healthy subjects were analyzed by a series of bioinformatics and statistical analyses. Two clusters of OP microbiotas in H7N9 patients, i.e., Cluster_1_Diseased and Cluster_2_Diseased, were determined at two microbial colonization states by Partition Around Medoids (PAM) clustering analysis, each characterized by distinct operational taxonomic units (OTUs) and functional metabolites. Cluster_1_Diseased was determined at more severe dysbiosis status compared with Cluster_2_Diseased, while OTU143_*Capnocytophaga* and OTU269_*Treponema* acted as gatekeepers for both of the two clustered microbiotas. Nine OTUs assigned to seven taxa, i.e., *Alloprevotella, Atopobium, Megasphaera, Oribacterium, Prevotella, Stomatobaculum*, and *Veillonella*, were associated with both H7N9 patients with and without secondary bacterial lung infection in Cluster_1. In addition, two groups of healthy cohorts may have potential different susceptibilities to H7N9 infection. These findings suggest that two OP microbial colonization states of H7N9 patients were at different dysbiosis states, which may help determine the health status of H7N9 patients, as well as the susceptibility of healthy subjects to H7N9 infection.

## Introduction

Avian influenza has caused great mortalities to human beings and animals during the last two decades (1–4). The highly pathogenic avian influenza A H7N9 virus emerged in eastern China in 2013 (5, 6). This virus has resulted in severe illness in the infected patients, including pneumonia and acute respiratory distress syndrome, with significant intensive care unit admission and a high case-fatality rate of 39% from 2013 to 2018 (7, 8).

Secondary bacterial lung infection (SBLI) is a usual condition in the H7N9 patients (9). *Acinetobacter baumanii, Candida albicans, Flavobacterium indologenes, Klebsiella, Pseudomonas*, and *Staphylococcus* have been isolated from blood, white blood cell, or sputum of H7N9 patients with SBLI (H7N9_SBLI), whereas no bacterium was isolated from those in the H7N9 patients without SBLI (H7N9_NSI) (10).

Oropharynx acts as one key gatekeeper of human airway (11) and the dysbiosis of oropharyngeal (OP) microbiotas are associated with multiple diseases (12–15). The disordered OP microbiotas were associated with H7N9_SBLI, with more abundant *Atopobium, Eubacterium, Leptotrichia, Oribacterium, Rothia, Solobacterium*, and *Streptococcus* in H7N9_SBLI than H7N9_NSI (10). Whether some bacteria could possibly contribute to the worse OP microbial colonization state(s) in both H7N9_SBLI or H7N9_NSI needs further investigations.

Diseased cohorts could have different microbial colonization states in the affected tissues or organs (16–18), which may reflect the severity or stages of disease and affect the effectiveness of the treatments. The OP microbial colonization states of the H7N9 patients remain poorly understood. The present study was designed to (1) determine the vital members and characteristics of different OP microbial colonization states in H7N9 patients; (2) investigate the bacteria that possibly drive the OP microbiotas to worse microbial dysbiosis state in both H7N9_NSI and H7N9_SBLI; and, in addition, (3) determine whether the healthy subjects could have potential different susceptibilities to H7N9 infection.

## Methods

### Patients and Sampling

The current study recruited a total of 72 OP samples from 72 individuals collected by Lu et al. (10), including 21 H7N9 patients with no SBLI (H7N9_NSI), 21 H7N9 patients with SBLI (H7N9_SBLI), and 30 healthy subjects. The selection criteria for the H7N9 patients and healthy subjects were provided in our previous study (10). Written informed consent was obtained from all patients and healthy subjects, and the study was approved by the institutional review board and ethics committee of the First Affiliated Hospital of Zhejiang University.

### Molecular Methods

The molecular experiments were conducted by Lu, et al. (10). Briefly, DNA extraction was performed by using a Qiagen Mini DNA extraction Kit (Qiagen Inc., Germany) as per manufacturer's instructions, with some slight modifications. DNA was then amplified by bacterial 16S rRNA primers targeting V3-V4 regions before being submitted for Illumina sequencing.

### Bioinformatics Analyses

The raw sequencing data generated from our previous work (10) were used for bioinformatics analyses in the present study. Paired-end read sequences were merged and quality filtered using USEARCH analysis tool (19). Sequences were chimera filtered and clustered into groups of operational taxonomic units (OTUs) with identity threshold ≥97% using the clustering pipeline UPARSE (19). Phylotypes were then classified using QIIME against SILVA database (20). Each sample was rarefied to 4,300 randomly selected reads per sample, consistent with the shallowest sample.

### Clustering of OP Microbiotas of All Individuals Based on Their Colonization States

The OP microbiotas in all the H7N9 and healthy subjects were clustered by Partition Around Medoids (PAM) clustering analysis in order to determine their microbial colonization states. Before PAM clustering, the average silhouette method was used to determine the optimal numbers of clusters for all the OP microbiotas (21).

Two clusters of OP microbiotas were determined in healthy cohorts (i.e., Cluster_1_Healthy and Cluster_2_Healthy) and H7N9 cohorts (i.e., Cluster_1_Diseased and Cluster_2_Diseased). A Pearson χ^2^ test was performed to compare the numbers of healthy and H7N9 cohorts in the two clusters.

### Comparisons of OP Microbiotas of H7N9 Patients in the Two Clusters

Permutation analysis of variance (PERMANOVA) was performed in R software version 3.6.1 with the vegan package (22) to determine the difference between Cluster_1_Diseased and Cluster_2_Diseased. Similarity percentage (SIMPER) analysis was conducted to identify the similarities within Cluster_1_Diseased and within Cluster_2_Diseased, as well as the dissimilarity of Cluster_1_Diseased and Cluster_2_Diseased. The richness (observed species), diversity (Shannon index), and evenness (Pielou index) of Cluster_1_Diseased and Cluster_2_Diseased were calculated and compared with *t* test.

### OTUs and Functional Metabolites Associated With the Two Clustered OP Microbiotas of H7N9 Patients

Linear discriminant analysis (LDA) effect size (LEfSe) was performed using Kruskal–Wallis test (α < 0.05), followed by a Wilcoxon rank-sum test (α < 0.05), and a one-against-all strategy for multiclass analysis (23). It was applied to identify the OTUs associated with Cluster_1_Diseased or Cluster_2_Diseased, with an LDA threshold >3.0. The functional metabolites of Cluster_1_Diseased or Cluster_2_Diseased were determined by using Tax4fun package in R software against SILVA database (24), followed by an LEfSe analysis to determine the functional metabolites associated with Cluster_1_Diseased or Cluster_2_Diseased.

### Bacterial Networks and Gatekeepers in the Two Clustered OP Microbiotas of H7N9 Patients

Co-occurrence Network (CoNet) analysis was carried out to investigate the co-occurrence and coexclusion of OTUs in Cluster_1_Diseased and Cluster_2_Diseased and to determine the top 10 OTUs with most correlations in the OP bacterial networks of Cluster_1_Diseased and Cluster_2_Diseased. The detailed processes followed the procedures described by Wagner Mackenzie et al. (25). Briefly, Pearson, Spearman, mutual information, Bray Curtis, and Kullback–Leibler dissimilarities were used to calculate the ensemble inference and the top 1,000 positive and negative correlations were recorded. The method-specific *P* values were computed by a permutation step, followed by a bootstrap procedure to merge the *P* values into one final *P* value using a method by Brown (26).

Gatekeepers were defined as the phylotypes interacting with different parts of the network to hold together the bacterial community (25, 27). In the present study, network fragmentation analysis was performed to identify the gatekeepers of OP bacterial networks of Cluster_1_Diseased and Cluster_2_Diseased. The detailed manipulations were performed as described by Wagner Mackenzie et al. (25). A null distribution of fragmentation scores was created from 10, 000 randomly constructed networks with identical node and edge distributions to the original network, and statistical significance was defined as the number of times a fragmentation score greater than the null fragmentation score resulting from the removal of the OTU observed within the null distribution.

### Dysbiosis Status of OP Microbiotas in Patients Within the Two Clusters

The OTUs differentiating the OP microbiotas of H7N9 patients from those of healthy subjects were determined by using the Galaxy implementation of LEfSe run by Huttenhower laboratory (23).

Dysbiosis ratios of bacterial taxa were associated with different diseases and conditions (28, 29). In the current study, avian influenza dysbiosis ratio (AIDR) was defined as the abundance ratio of OTUs associated with healthy cohort and OTUs associated with H7N9, to assist in determining the dysbiosis states of OP microbiotas in the H7N9 cohorts within the two clusters. Avian influenza dysbiosis ratios of the healthy and H7N9 cohorts were transformed in log10 to satisfy the assumptions of normal distribution and equal variance before being compared with a *t* test. The same data transformation and statistical approach were applied for the comparison of the AIDRs of Cluster_1_Diseased and Cluster_2_Diseased.

A series of statistical analyses were also performed to help determine the dysbiosis status of the two clusters of OP microbiotas in H7N9 patients. Mann–Whitney *U* test was used to compare the abundances of the OTUs associated with H7N9 between Cluster_1_Diseased and Cluster_2_Diseased. The same approach was performed for the comparisons of OTUs associated with healthy cohort between Cluster_1_Diseased and Cluster_2_Diseased. A Pearson χ^2^ test was applied to compare the numbers of OTUs that were associated with H7N9 and more abundant in Cluster_1_Diseased or Cluster_2_Diseased. The same test was carried out for the comparisons of the numbers of OTUs, which were associated with healthy cohort and more abundant in Cluster_1_Diseased or Cluster_2_Diseased.

### Comparisons of OP Microbiotas in H7N9_NSI or H7N9_SBLI Between Cluster_1 and Cluster_2

The two clustered OP microbiotas in H7N9_NSI (or H7N9_SBLI) were compared to determine whether some OTUs could possibly drive the OP microbiotas to worse microbial dysbiosis state in both H7N9_NSI and H7N9_SBLI.

Avian influenza dysbiosis ratios of H7N9_NSI cohorts in the two clusters were compared by a *t* test, after being transformed in log10 to satisfy the assumptions of normal distribution and equal variance. The same approaches were applied for the comparisons of the AIDRs of H7N9_SBLI cohorts in the two clusters.

An LEfSe analysis was applied to determine the OTUs differentiating the two clustered OP microbiotas in H7N9_NSI (i.e., Cluster_1_H7N9_NSI and Cluster_2_H7N9_NSI). The same approach was carried out for determining the OTUs associated with each of the two clustered OP microbiotas in H7N9_SBLI (i.e., Cluster_1_H7N9_SBLI or Cluster_2_H7N9_SBLI).

Similarly, an LEfSe analysis was used to determine the functional metabolites associated with Cluster_1_H7N9_NSI or Cluster_2_H7N9_NSI. The same analysis was used to determine the functional metabolites associated with Cluster_1_H7N9_SBLI or Cluster_2_H7N9_SBLI.

The OTUs associated with both Cluster_1_H7N9_NSI and Cluster_1_H7N9_SBLI were determined by an online program Venny diagram version 2.1 (30). The same program was used to determine the OTUs associated with both Cluster_2_H7N9_NSI and Cluster_2_H7N9_SBLI.

### Comparisons of the Two Clustered Healthy OP Microbiotas

Permutation analysis of variance was applied to determine the difference between Cluster_1_Healthy and Cluster_2_Healthy. Avian influenza dysbiosis ratios of Cluster_1_Healthy and Cluster_2_Healthy were transformed in log10 to satisfy the assumptions of normal distribution and equal variance, before being compared by a *t* test.

An LEfSe analysis was applied to determine the OTUs associated with each of the two clustered OP microbiotas in healthy cohorts. The same approach was used for identifying the functional metabolites associated with Cluster_1_Healthy or Cluster_2_Healthy.

## Results

### Clustering of OP Microbiotas in All Individuals

**Figure 1:**
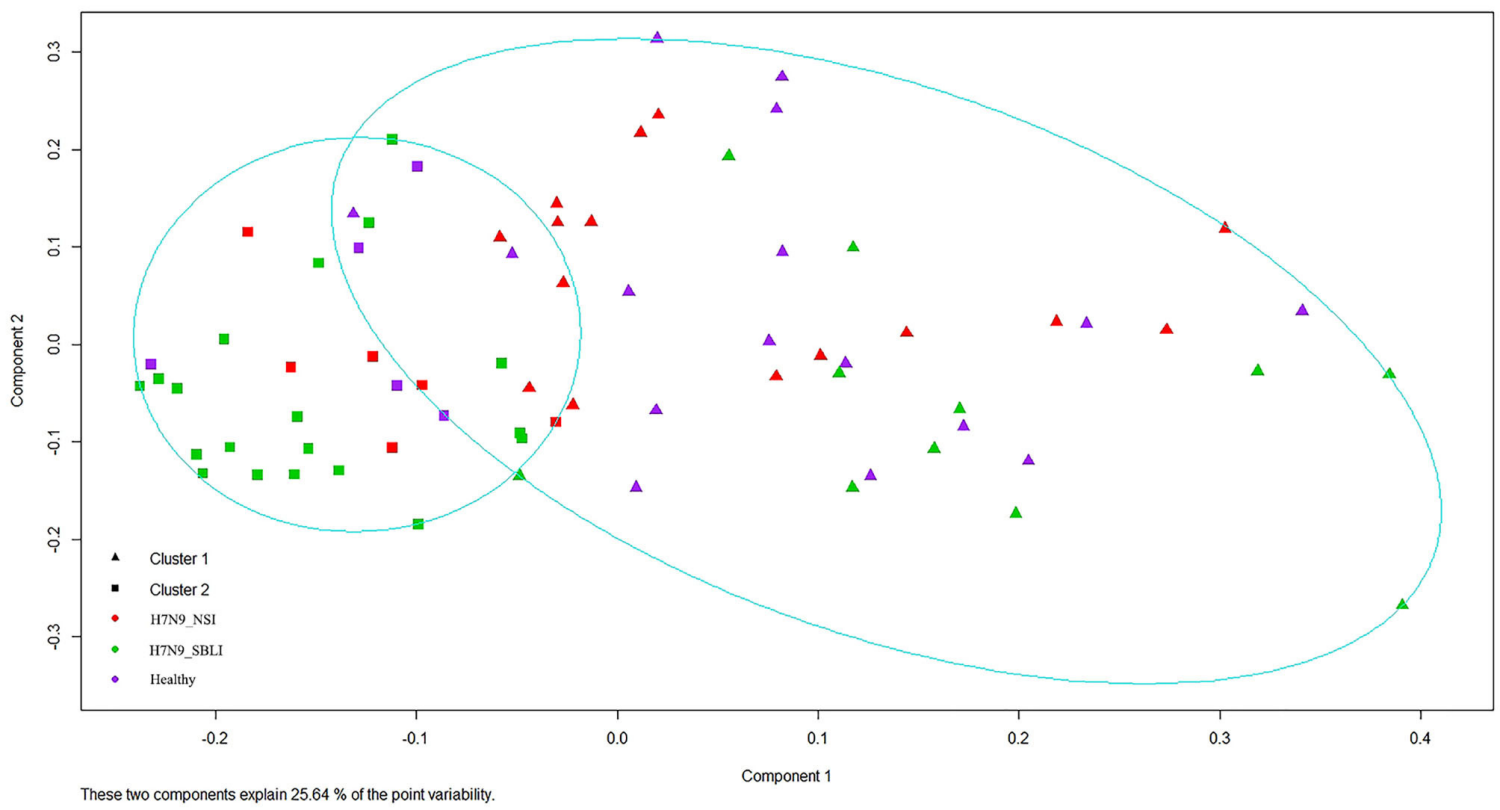
All the 72 oropharyngeal (OP) microbiotas were clustered into two clusters by Partition Around Medoids (PAM) clustering analysis.

Silhouette analysis identified two as the most optimal number for clustering all the 72 OP microbiotas (Figure S1). Therefore, the OP microbiotas of all individuals were clustered into two clusters (i.e., Cluster_1 and Cluster_2) by PAM clustering analysis (Figure 1). Cluster_1 included a total of 31 H7N9 microbiotas (Cluster_1_Diseased) and 11 healthy microbiotas (Cluster_1_Healthy), whereas Cluster_2 contained 11 H7N9 microbiotas (Cluster_2_Diseased) and 19 healthy microbiotas (Cluster_2_Healthy) (Figure 1). There was a significant difference between the numbers of OP microbiotas from H7N9 or healthy cohorts in the two clusters (χ^2^ = 9.933, *P* = 0.002).

### Differences Between Cluster_1_Diseased and Cluster_2_Diseased

The five most abundant phyla in the OP microbiotas of H7N9 patients accounted for >90% abundance of the OP microbiotas. Among them, *Bacteroidetes, Fusobacteria*, and *Proteobacteria* were more abundant in Cluster_2_Diseased compared with Cluster_1_Diseased, whereas *Firmicutes* and *Saccharibacteria* were more abundant in Cluster_1_Diseased than in Cluster_2_Diseased. Nine most abundant orders in the H7N9 patients constituted >90% abundance of all the orders in H7N9 microbiotas, among which *Campylobacterales, Clostridiales, Flavobacteriales, Lactobacillales*, and *Selenomonadales* had greater abundances in Cluster_1_Diseased compared with Cluster_2_Diseased, whereas *Bacteroidales, Fusobacteriales, Neisseriales*, and *Pasteurellales* were more abundant in Cluster_2_Diseased than in Cluster_1_Diseased.

Permutation analysis of variance determined that Cluster_1_Diseased and Cluster_2_Diseased were significantly different (*R*^2^ = 0.090, *P* < 0.001). The dissimilarity between Cluster_1_Diseased and Cluster_2_Diseased was relatively high (SIMPER dissimilarity = 64.8%) according to SIMPER results. The similarity within Cluster_1_Diseased (SIMPER average similarity = 38.2%) was lower than that within Cluster_2_Diseased (SIMPER average similarity = 44.1%). Both richness and diversity were similar in Cluster_1_Diseased and Cluster_2_Diseased (*t* test, all *P* > 0.07), but the evenness was significantly higher in Cluster_1_Diseased than in Cluster_2_Diseased (*t* test, *P* < 0.05) (Table 1).

**Table 1:** Comparisons of richness (observed species), diversity (Shannon), and evenness (Pielou) of two clustered oropharyngeal (OP) microbiotas in H7N9 patients (i.e., Cluster_1_Diseased and Cluster_2_Diseased).

**Alpha diversity indices**	**Cluster_1_Diseased (mean ± SE)**	**Cluster_2_Diseased (mean ± SE)**
Observed species	296 ± 51	282 ± 27
Shannon	4.37 ± 0.06	4.18 ± 0.06
Pielou	0.77 ± 0.007^*^	0.74 ± 0.009^*^

* *Significant difference with P < 0.05*.

**Figure 2:**
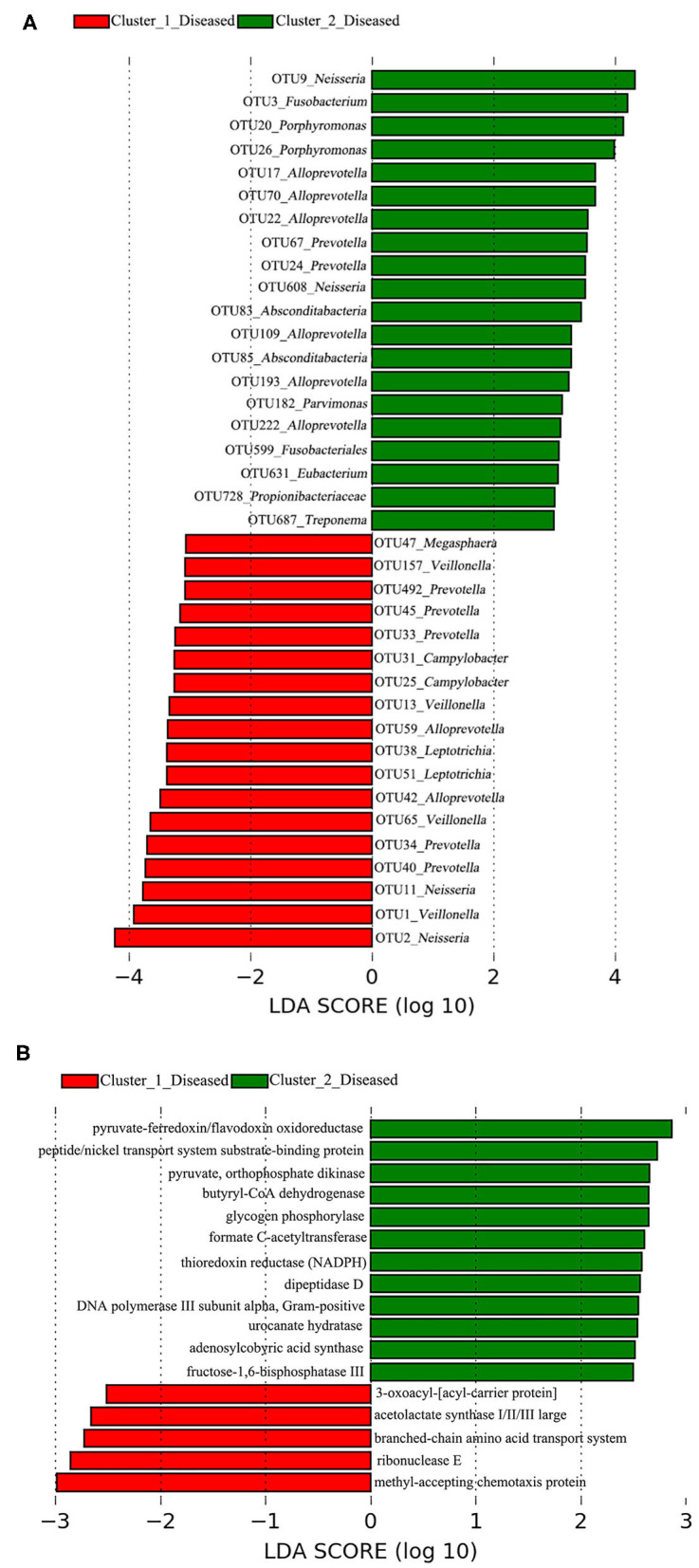
Linear discriminant analysis (LDA) effect size (LEfSe) determined the **(A)** operational taxonomic units (OTUs) and **(B)** functional metabolites associated with each of the two clustered OP microbiotas of H7N9 patients.

A total of 38 OTUs were identified differentiating Cluster_1_Diseased and Cluster_2_Diseased, among which OTU2 and OTU9 (both assigned to *Neisseria*) with the largest LDA scores were closely associated with Cluster_1_Diseased and Cluster_2_Diseased, respectively (Figure 2A). Likewise, a range of 17 functional metabolites were determined capable of differentiating Cluster_1_Diseased and Cluster_2_Diseased (Figure 2B). Methyl-accepting chemotaxis protein was the leading metabolite differentiating Cluster_1_Diseased from Cluster_2_Diseased.

### Dysbiosis Status of Cluster_1_Diseased and Cluster_2_Diseased

The LEfSe results showed that 69 OTUs were associated with H7N9, and 22 OTUs were associated with healthy cohort, which were used for the calculations and comparisons of AIDRs in different groups (Table S1). The healthy subjects have a significantly greater AIDR (14.27 ± 2.83 SE) than that of H7N9 patients (2.48 ± 0.41 SE) (*t* test, *P* < 0.001), suggesting a lower AIDR was likely to indicate the dysbiosis of OP microbiotas in H7N9 patients compared with healthy subjects. The AIDR was significantly higher in Cluster_2_Diseased (5.28 ± 1.12 SE) than Cluster_1_Diseased (1.48 ± 0.21 SE) (*t* test, *P* < 0.001).

**Figure 3:**
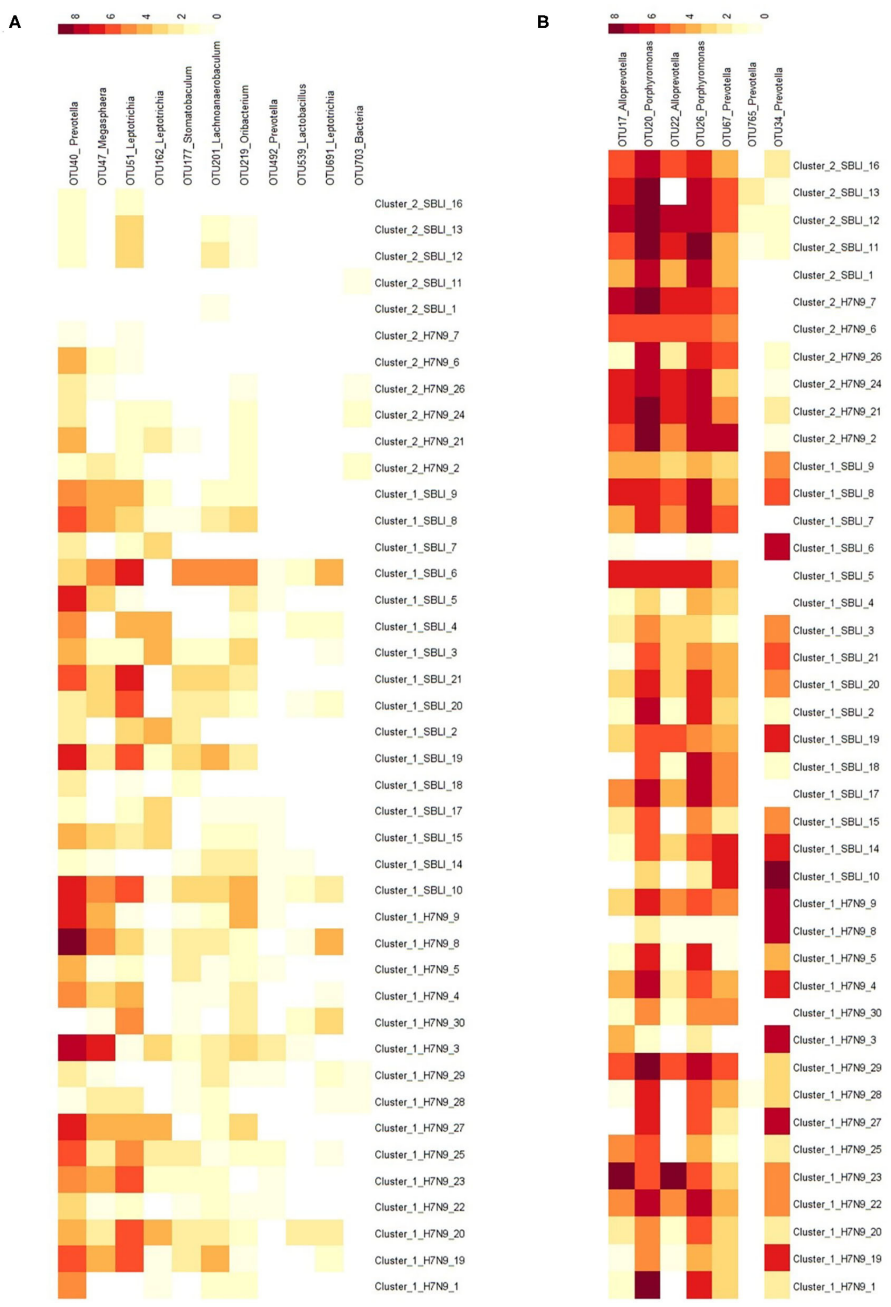
Distributions of the OTUs associated with **(A)** H7N9 or **(B)** healthy cohort in the two clustered H7N9 patients. The demonstrated OTUs had significantly different abundances between the two clustered H7N9 patients.

Eleven (of 69) OTUs associated with H7N9 were determined with significantly different abundances between Cluster_1_Dieased and Cluster_2_Diseased (Mann–Whitney test, all *P* < 0.05) (Figure 3A). Among them, significantly more OTUs (10 OTUs) associated with H7N9 were more abundant in Cluster_1_Diseased than in Cluster_2_Diseased (χ^2^ = 11.0, *P* = 0.001).

Likewise, seven (of 22) OTUs associated with healthy cohort were determined with significantly different abundances between the two clustered H7N9 microbiotas (Mann–Whitney test, all *P* < 0.02) (Figure 3B). Among them, significantly more OTUs (six OTUs) associated with healthy cohort were more abundant in Cluster_2_Diseased than in Cluster_1_Diseased (χ^2^ = 7.0, *P* < 0.01).

These above results consistently suggested that Cluster_2_Diseased were at better dysbiosis status compared with Cluster_1_Diseased.

### Networks and Gatekeepers in Cluster_1_Diseased and Cluster_2_Diseased

The two bacterial networks of Cluster_1_Diseased and Cluster_2_Diseased were determined by CoNet analysis (Figure S2). None of the top 10 OTUs with the most correlations was found in the networks of both Cluster_1_Diseased and Cluster_2_Diseased (Table 2).

**Table 2:** The top 10 OTUs with most correlations in the OP bacterial networks of the two clustered OP microbiotas of H7N9 patients.

	**Cluster_1_Diseased**		**Cluster_2_Diseased**	
**Rank**	**OTU ID**	**Taxonomy**	**OTU ID**	**Taxonomy**
1	33	*Prevotella*	149	*Alloprevotella*
2	108	*Filifactor*	142	*Prevotella*
3	122	*Streptococcus*	431	*Rikenellaceae*
4	19	*Porphyromonas*	251	*Parvimonas*
5	438	*Capnocytophaga*	310	*Parvimonas*
6	133	*Mycoplasma*	159	*Fretibacterium*
7	34	*Prevotella*	622	*Corynebacterium*
8	510	*Prevotella*	86	*Absconditabacteria*
9	45	*Prevotella*	279	*Prevotella*
10	160	*Megasphaera*	118	*Prevotella*

Fragmentation results demonstrated a lower fragmentation score in Cluster_2_Diseased (i.e., 0.419) than that of Cluster_1_Diseased (i.e., 0.717), suggesting that Cluster_2_Diseased had stronger co-occurrence patterns and greater biotic interactions than Cluster_1_Diseased. A group of nine OTUs was determined to be gatekeepers of Cluster_1_Diseased, and another group of nine OTUs was identified as gatekeepers of Cluster_2_Diseased (Table 3). OTU143_*Capnocytophaga* and OTU269_*Treponema* were determined as gatekeepers for both Cluster_1_Diseased and Cluster_2_Diseased.

**Table 3:** Gatekeepers in the two OP bacterial networks of the two clustered H7N9 microbiotas identified by fragmentation analysis.

**Cluster_1_Diseased**		**Cluster_2_Diseased**	
**OTU ID**	**Taxonomy**	**OTU ID**	**Taxonomy**
58	*Capnocytophaga*	7	*Fusobacterium*
66	*Prevotella*	143^*^	*Capnocytophaga*
143^*^	*Capnocytophaga*	149	*Alloprevotella*
165	*Saccharibacteria*	206	*Fusobacterium*
229	*Gemella*	269^*^	*Treponema*
237	*Treponema*	282	*Neisseria*
269^*^	*Treponema*	377	*Lentimicrobiaceae*
370	*Fusobacterium*	413	*Prevotella*
554	*Prevotella*	544	*Prevotella*

* *The OTUs commonly found as gatekeepers in the two clustered OP microbiotas in H7N9 patients*.

### Comparisons of Two Clustered OP Microbiotas in H7N9_NSI or H7N9_SBLI

The two clustered OP microbiotas in H7N9_NSI or H7N9_SBLI were compared to determine the bacteria that could possibly drive the OP microbiotas to more severe microbial dysbiosis state in both H7N9_NSI and H7N9_SBLI.

Avian influenza dysbiosis ratio was significantly higher in Cluster_2_H7N9_NSI (5.98 ± 1.51 SE) compared with Cluster_1_H7N9_NSI (1.43 ± 0.31 SE) (*t* test, *P* = 0.001). Likewise, Cluster_2_H7N9_SBLI (4.44 ± 1.76 SE) had a significantly higher AIDR compared with Cluster_1_H7N9_SBLI (1.53 ± 0.30 SE) (*t* test, *P* < 0.03).

**Figure 4:**
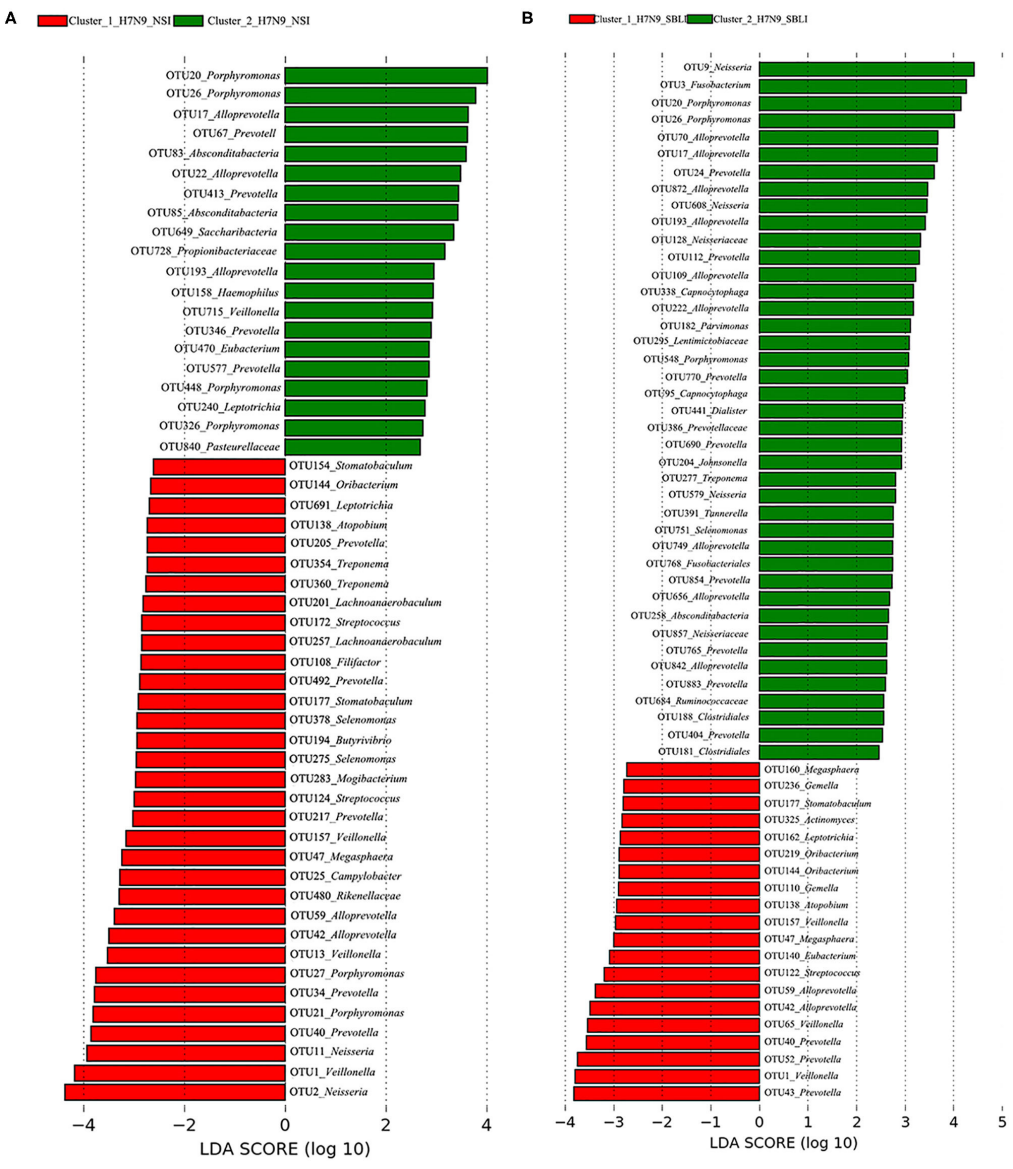
Operational taxonomic units associated with each of the two clustered OP microbiotas of **(A)** H7N9 patients with no secondary bacterial lung infection (H7N9_NSI) and **(B)** H7N9 patients with secondary bacterial lung infection (H7N9_SBLI).

**Figure 5:**
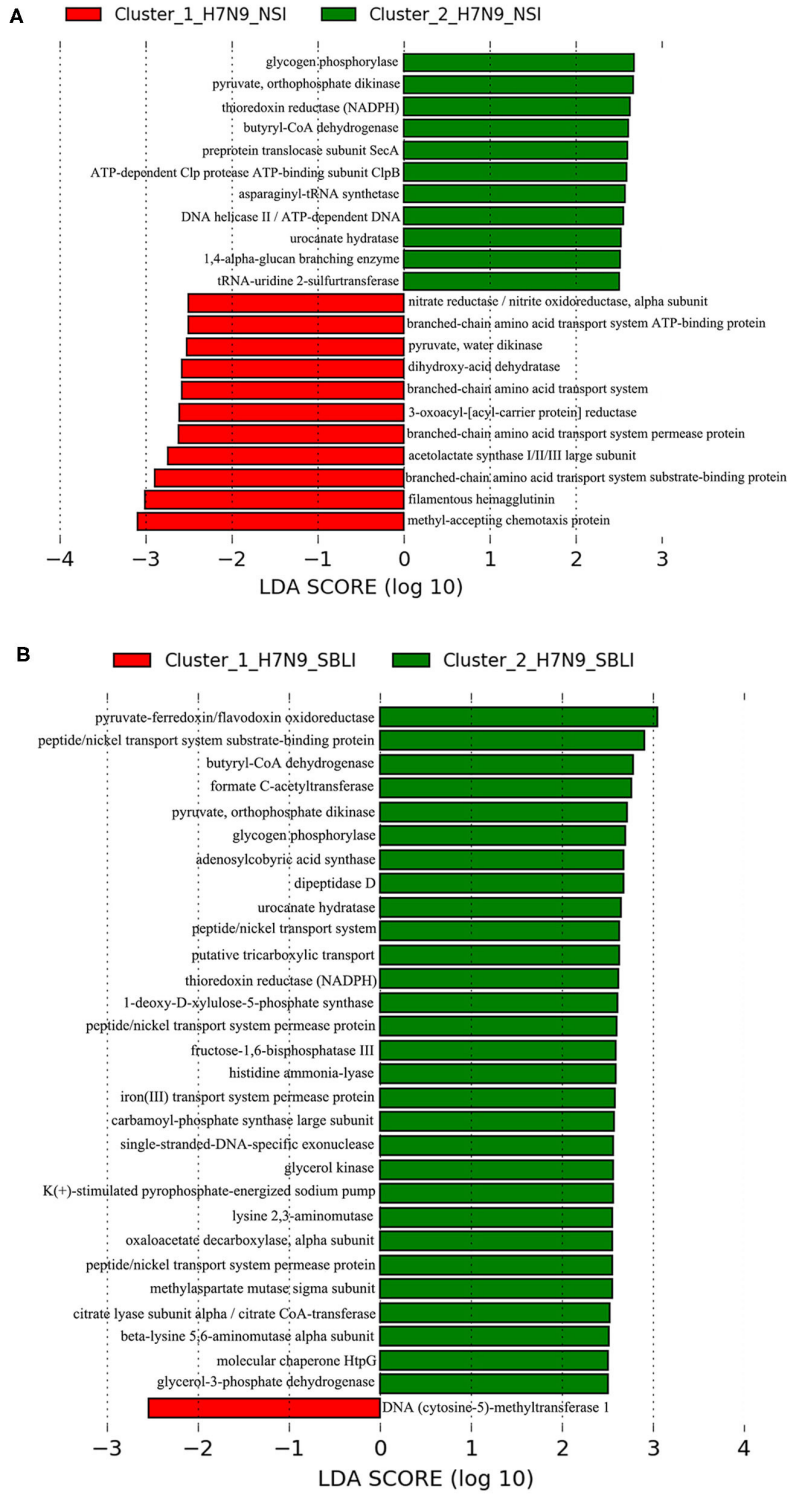
Functional metabolites associated with each of the two clustered OP microbiotas of **(A)** H7N9_NSI and **(B)** H7N9_SBLI.

LEfSe results showed that 33 OTUs were associated with OP microbiotas of H7N9_NSI in Cluster_1 (Cluster_1_H7N9_NSI), and 20 OTUs were associated with OP microbiotas of H7N9_NSI in Cluster_2 (Cluster_2_H7N9_NSI) (Figure 4A). A total of 22 functional metabolites were determined to differentiate the Cluster_1_H7N9_NSI and Cluster_2_H7N9_NSI (Figure 5A).

Likewise, a total of 20 OTUs were more associated with Cluster_1_H7N9_SBLI, and 41 OTUs were more associated with Cluster_2_H7N9_SBLI (Figure 4B). A total of 30 functional metabolites were determined differentiating Cluster_1_H7N9_SBLI and Cluster_2_H7N9_SBLI, most of which (29) were more associated with Cluster_1_H7N9_SBLI, whereas only DNA (cytosine-5)-methyltransferase 1 was more associated with Cluster_2_H7N9_SBLI (Figure 5B).

**Figure 6:**
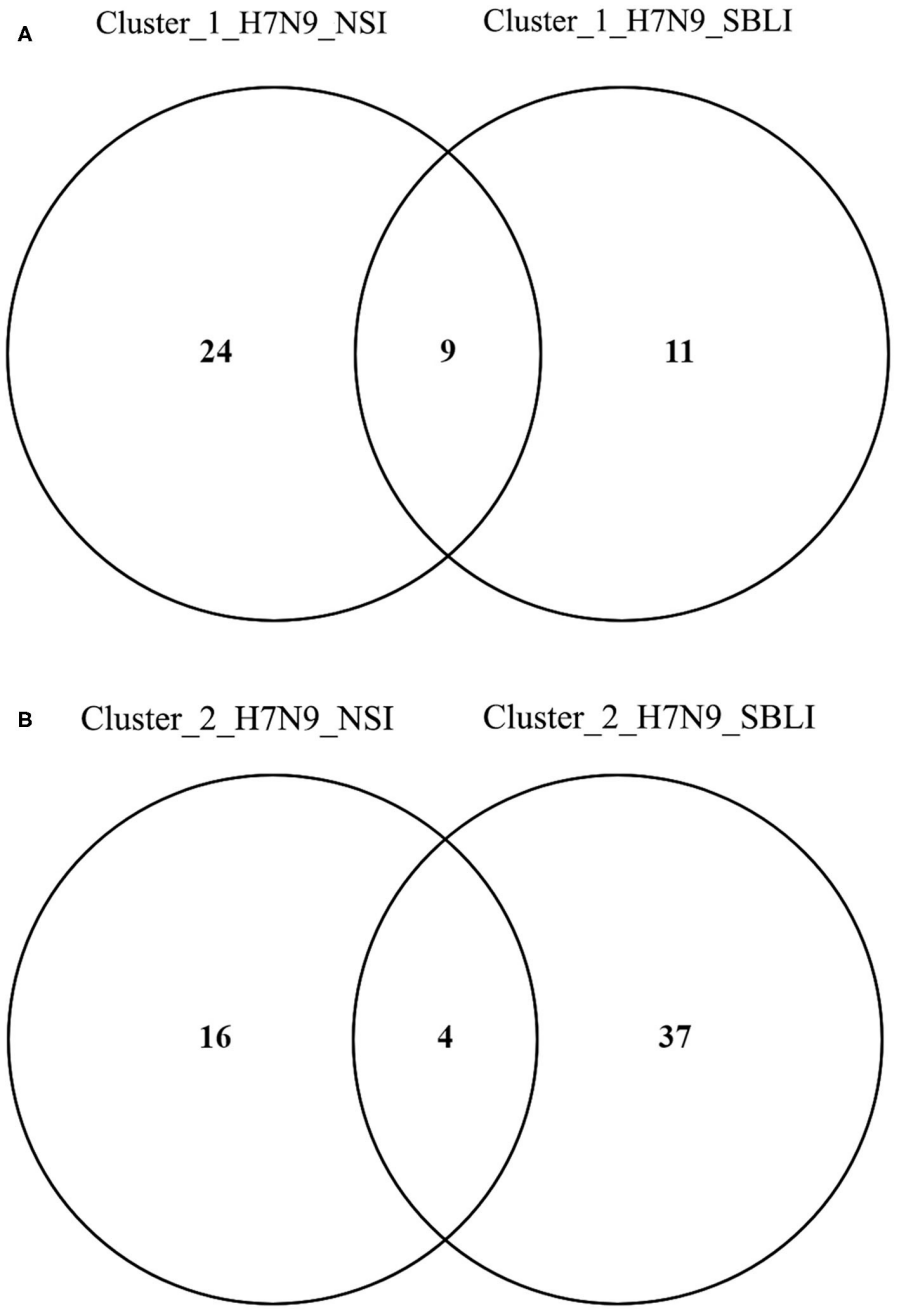
Operational taxonomic units associated with **(A)** OP microbiotas of both H7N9_NSI and H7N9_SBLI in Cluster_1 (i.e., Cluster_1_H7N9_NSI and Cluster_1_H7N9_SBLI) and **(B)** OP microbiotas of both H7N9_NSI and H7N9_SBLI in Cluster_2 (i.e., Cluster_2_H7N9_NSI and Cluster_2_H7N9_SBLI).

More importantly, there were nine OTUs (i.e., OTUs 1, 40, 42, 47, 59, 138, 144, 157, and 177) associated with both Cluster_1_H7N9_NSI and Cluster_1_H7N9_SBLI (Figure 6A), which were assigned to seven taxa, including *Alloprevotella, Atopobium, Megasphaera, Oribacterium, Prevotella, Stomatobaculum*, and *Veillonella*. Likewise, four OTUs (i.e., 17, 20, 26, and 193) assigned to *Alloprevotella* or *Porphyromonas* were associated with both Cluster_2_H7N9_NSI and Cluster_2_H7N9_SBLI (Figure 6B).

In addition, five of the 20 OTUs (i.e., OTUs 17, 20, 22, 26, and 67) associated with Cluster_2_H7N9_NSI were also identified being associated with healthy cohort. Likewise, five of 41 OTUs (i.e., OTUs 17, 20, 26, 749, and 765) associated with Cluster_2_H7N9_SBLI were also determined being associated with healthy cohort.

### Correlations of the OTUs Associated With H7N9_NSI or H7N9_SBLI in Two Clusters

**Figure 7:**
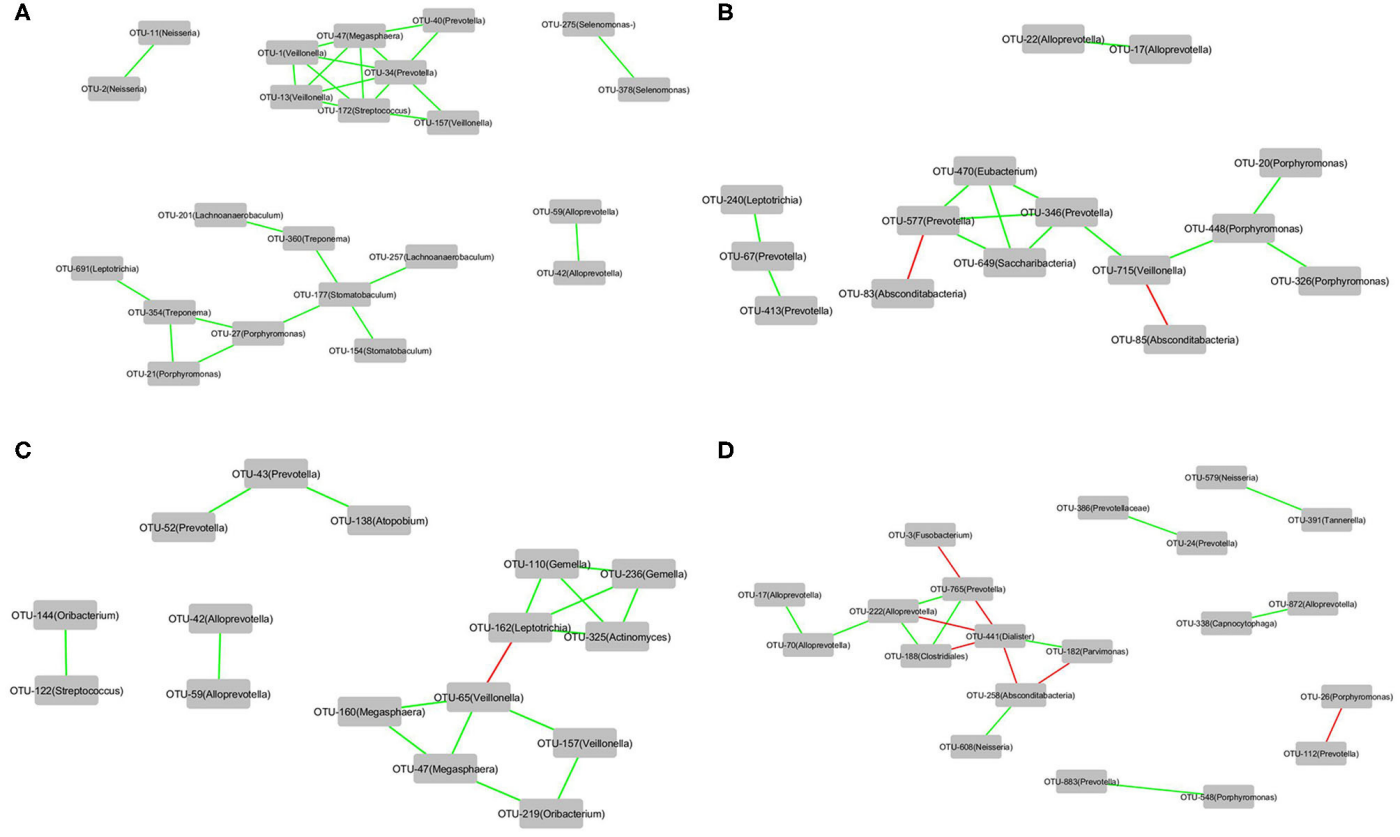
The correlations of the OTUs that were associated with **(A)** Cluster_1_H7N9_NSI, **(B)** Cluster_2_H7N9_NSI, **(C)** Cluster_1_H7N9_SBLI, and **(D)** Cluster_2_H7N9_SBLI.

The correlations of the OTUs associated with each of the four groups, that is, Cluster_1_H7N9_NSI, Cluster_2_H7N9_NSI, Cluster_1_H7N9_SBLI, and Cluster_2_H7N9_SBLI, were determined by CoNet analyses (Figure 7). OTU441_*Dialister* was negatively correlated with four other OTUs associated with Cluster_2_H7N9_SBLI (Figure 7D), whereas most of the other OTUs had positive correlations in the four bacterial networks.

### Differences Between Cluster_1_Healthy and Cluster_2_Healthy

The PERMANOVA results showed a significant difference between the Cluster_1_Healthy and Cluster_2_Healthy (*R*^2^ = 0.689, *P* < 0.001). Avian influenza dysbiosis ratio was significantly higher in Cluster_2_Healthy (19.74 ± 3.83 SE) than Cluster_1_Healthy (4.83 ± 1.91 SE) (*t* test, *P* < 0.001).

**Figure 8:**
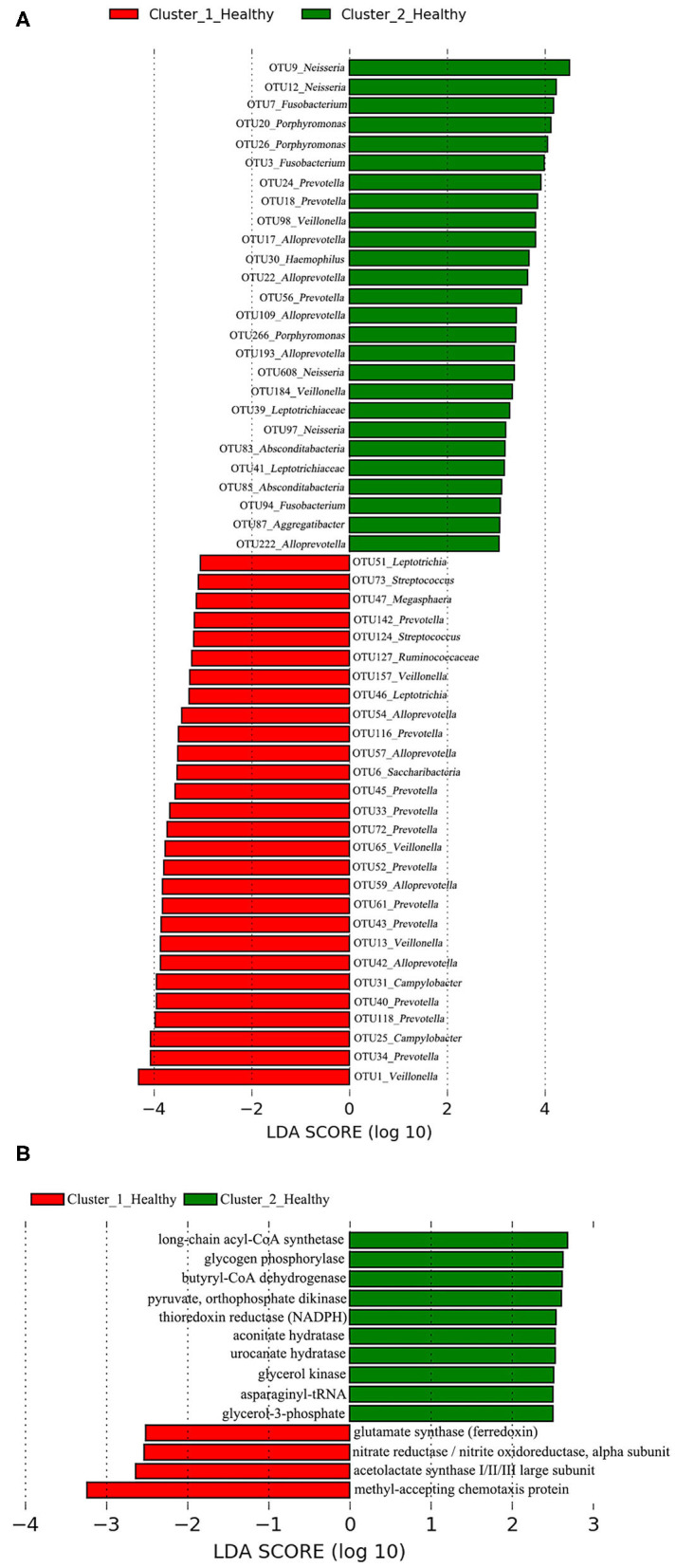
LEfSe analyses determined the **(A)** OTUs and **(B)** functional metabolites associated with each of the two clustered OP microbiotas of healthy subjects.

A total of 28 OTUs were associated with Cluster_1_Healthy, with OTU1_*Veillonella*, OTU34_*Prevotella*, and OTU25_*Campylobacter* as the top three phylotypes differentiating Cluster_1_Healthy from Cluster_2_Healthy (Figure 8A). Likewise, 26 OTUs were associated with Cluster_2_Healthy, among which OTU9_*Neisseria*, OTU12_*Neisseria*, OTU7_*Fusobacterium*, OTU20_*Porphyromonas*, and OTU26_*Porphyromonas* were the leading phylotypes differentiating Cluster_2_Healthy from Cluster_1_Healthy (Figure 8A).

Four and 10 functional metabolites were associated with Cluster_1_Healthy and Cluster_2_Healthy, respectively (Figure 8B). The methyl-accepting chemotaxis protein was the metabolite most associated with Cluster_1_Healthy. By contrast, long-chain acyl-CoA synthetase, glycogen phosphorylase, and butyryl-CoA dehydrogenase were the three metabolites most associated with Cluster_2_Healthy.

## Discussion

Highly pathogenic avian influenza virus, that is, H5N1, H5N6, and H7N9, could cause mortalities in human beings (3, 4, 7, 31–34). H7N9 has attracted great scientific interests since its emergence from the year 2013 (4, 35–42). However, there is a lack of research on the microbiotas in H7N9 patients (10, 43).

Oropharyngeal microbiota was believed as an important source of lung microbiota in human adults and children (11, 44, 45). Disordered OP microbiota was associated with multiple human diseases, such as schizophrenia, chronic obstructive pulmonary disease, and OP, head, and neck cancers (46–49). Different microbial colonization states have been determined on the affected tissues or organs of diseased cohorts (16–18). Different OP microbial colonization states could be associated with the stages or severities of disease and affect the effectiveness of the treatments. In the current study, we explored the vital members and characteristics of different OP microbial colonization states in H7N9 patients.

### Comparisons of Two OP Microbial Colonization States in H7N9 Patients

Partition Around Medoids clustering analysis has been used to cluster the bacterial communities in different disease studies (16–18, 50, 51). In the present study, the OP microbiotas of both H7N9 and healthy cohorts were clustered into two clusters by PAM analysis. The significantly greater presence of H7N9 patients in Cluster_1 than in Cluster_2 suggested that OP microbiotas in the H7N9 patients within Cluster_1 were at worse dysbiosis status than those within Cluster_2.

Great difference was determined between Cluster_1_Diseased and Cluster_2_Diseased according to the PERMANOVA and SIMPER results, as well as the different evenness, suggesting that the two OP microbial colonization states in H7N9 patients were largely different.

Dysbiosis ratios of bacterial taxa are associated with different diseases and conditions (28, 52–54). For example, cirrhosis dysbiosis ratio was associated with the health status of cirrhotic cohorts (29). In the current study, AIDR was used to help determine the dysbiosis status of the two clustered OP microbiotas of H7N9 patients. Significantly lower AIDR in OP microbiotas of H7N9 patients than healthy subjects suggested AIDR is associated with the more severe dysbiosis status of OP microbiotas in H7N9 patients at baseline.

Avian influenza dysbiosis ratio was significantly higher in Cluster_2_Diseased than in Cluster_1_Diseased, suggesting the Cluster_2_Diseased was at better dysbiosis status compared with Cluster_1_Diseased. Less OTUs associated with H7N9 and more OTUs associated with healthy cohort were more abundant in Cluster_2_Diseased than in Cluster_1_Diseased, further suggesting that Cluster_2_Diseased was at better dysbiosis status compared with Cluster_1_Diseased.

*Neisseria* was determined as both pathogenic and non-pathogenic bacteria (55). In the present study, OTU2_*Neisseria* and OTU9_*Neisseria* were most closely associated with Cluster_1_Diseased and Cluster_2_Diseased, respectively, suggesting the OTUs with the same taxon could play different roles in the two clustered OP microbiotas of H7N9 patients. *Veillonella* was more abundant in the gut of H7N9 patients with or without antibiotics compared with healthy controls (43). In the current study, four *Veillonella* phylotypes (i.e., OTUs 1, 65, 13, and 157) were associated with Cluster_1_Diseased, whereas no *Veillonella* was associated with Cluster_2_Diseased, suggesting that the increased *Veillonella* in the OP microbiota could be a source of *Veillonella* in the gut of H7N9 patients with Cluster_1_Diseased.

Fragmentation analysis has been performed to determine the fragmentation levels and gatekeepers of microbiotas in some disease studies (16, 25). In the present study, lower level of network fragmentation was found in Cluster_2_Diseased than Cluster_1_Diseased, suggesting the Cluster_2_Diseased had stronger co-occurrence patterns and increased biotic interactions, implying the bacterial network of Cluster_1_Diseased may experience greater interruptions. *Capnocytophaga* was part of resident OP microbiota in human and could be opportunistic pathogens of extraoral infections (56–59), whereas *Treponema* was associated with endodontic infections (60, 61). Among the gatekeepers for Cluster_1_Diseased and Cluster_2_Diseased, OTU143_*Capnocytophaga* and OTU269_*Treponema* were determined as gatekeepers for both the two clustered microbiotas, suggesting the two phylotypes could possibly maintain the OP bacterial networks for consistent H7N9 infections in the recruited patients.

### Comparisons of H7N9_NSI and H7N9_SBLI in the Two Clusters

The OP microbiotas of H7N9_NSI and H7N9_SBLI in Cluster_2 were both determined at better dysbiosis status compared with those in Cluster_1 according to the AIDR comparison results, suggesting both Cluster_2_H7N9_NSI and Cluster_2_H7N9_SBLI contributed to the better dysbiosis status of Cluster_2_Diseased compared with Cluster_1_Diseased.

*Atopobium* and *Oribacterium* could differentiate the OP microbiotas in H7N9_SBLI from those in H7N9_NSI (10). The current study revealed some phylotypes assigned to *Atopobium* and *Oribacterium* were associated with worse OP microbial colonization states (i.e., Cluster_1_H7N9_NSI and Cluster_1_H7N9_SBLI), suggesting these phylotypes were likely to cause more severe conditions in H7N9 patients. Some *Alloprevotella* and *Porphyromonas* species were determined as opportunistic oral pathogens (62–64). In the present study, OTU17_*Alloprevotella*, OTU20_*Porphyromonas*, OTU26_*Porphyromonas*, and OTU193_*Alloprevotella* were associated with both Cluster_2_H7N9_NSI and Cluster_2_H7N9_SBLI, suggesting the four phylotypes were more likely as opportunistic pathogens for inducing H7N9_NSI or H7N9_SBLI.

### Correlations of the OTUs Associated With H7N9_NSI or H7N9_SBLI in the Two Clusters

A few *Dialister* species were associated with some oral diseases, such as periodontal disease, apical periodontitis, and dentinal caries (65–67). Among the OTUs associated with Cluster_2_H7N9_SBLI, OTU441_*Dialister* was negatively correlated with four OTUs in Cluster_2_H7N9_SBLI, suggesting this phylotype was more likely as pathogenic bacteria and had competitive interactions with some other phylotypes within this OP microbial colonization state. We acknowledge that further studies are needed to confirm it.

### Functional Metabolites Associated With Each of the Two OP Microbial Colonization States in H7N9 Patients

Methyl-accepting chemotaxis protein is vital to the cell survival, pathogenesis, and biodegradation (68). In the current study, increased levels of methyl-accepting chemotaxis protein were more associated with both Cluster_1_Diseased and Cluster_1_H7N9_NSI compared with Cluster_2_Diseased and Cluster_2_H7N9_NSI, suggesting this metabolite was associated with worse dysbiosis state of OP microbiotas in H7N9 patients, possibly via enhancing the bacterial pathogenicity. Overexpression of DNA (cytosine-5)-methyltransferase 1 is associated with multiple diseases (i.e., psychosis vulnerability, liver, and stomach cancers) and plays a critical role in the malignant progression of hepatocellular carcinomas (69–71). DNA (cytosine-5)-methyltransferase 1 was the only functional metabolite differentiating Cluster_1_H7N9_SBLI from Cluster_2_H7N9_SBLI, suggesting this metabolite was commonly associated with the SBLI caused by varied bacteria during H7N9 infection.

### Potential H7N9-Susceptible and H7N9-Tolerant Healthy Subjects

Greater AIDR was determined in Cluster_2_Healthy than Cluster_1_Healthy, suggesting the healthy subjects in Cluster_2 could be more tolerant to H7N9 infection than those in Cluster_1. *Veillonella* was more abundant in the gut of H7N9 patients than healthy controls as described above (43), whereas methyl-accepting chemotaxis protein plays a vital role in bacterial pathogenicity as described above (68). In the present study, OTU1_*Veillonella* and methyl-accepting chemotaxis protein were determined as the phylotype and functional metabolite most associated with Cluster_1_Healthy, suggesting they were more likely to enhance the greater susceptibility of the healthy subjects to H7N9 infection.

In conclusion, two OP microbial colonization states were determined in the H7N9 patients, each characterized by distinct phylotypes. Nine phylotypes assigned to *Alloprevotella, Atopobium, Megasphaera, Oribacterium, Prevotella, Stomatobaculum*, and *Veillonella* were likely to drive the OP microbiotas to worse microbial dysbiosis state in both H7N9_NSI and H7N9_SBLI. In addition, healthy subjects could have different susceptibilities to H7N9 infection.

## Data Availability Statement

The raw sequencing data were deposited in NCBI under BioProject accession no. PRJNA638222.

## Ethics Statement

The studies involving human participants were reviewed and approved by The Institutional Review Board and Ethics Committee of the First Affiliated Hospital of Zhejiang University. The patients/participants provided their written informed consent to participate in this study.

## Author Contributions

HZha and LL designed the study. HL and HZhang collected samples and provided the raw sequencing data. HZha, JW, and KC contributed to the data analyses. HZha interpreted the results and drafted the manuscript. HZ, HL, and JW reviewed the manuscript. QW, JL, QL, and YL contributed to the literature search and participate in the study design. All authors approved the final manuscript.

## Conflict of Interest

The authors declare that the research was conducted in the absence of any commercial or financial relationships that could be construed as a potential conflict of interest.
